# The role of MRI in the prenatal diagnosis and classification of fetal microtia

**DOI:** 10.1007/s00330-023-09816-5

**Published:** 2023-06-14

**Authors:** Xiaodan Zhang, Weizeng Zheng, Yan Feng, Na Yu, Jiale Qin, Kui Li, Guohui Yan, Yu Zou, Baohua Li

**Affiliations:** 1 Department of Radiology, Women’s Hospital, Zhejiang University School of Medicine, Xueshi Rd. No.1, Hangzhou, China; 2 Department of Obstetrics, Women’s Hospital, Zhejiang University School of Medicine, Xueshi Rd. No.1, Hangzhou, China; 3 Department of Ultrasound, Women’s Hospital, Zhejiang University School of Medicine, Xueshi Rd. No.1, Hangzhou, China

**Keywords:** Prenatal diagnosis, Magnetic resonance imaging, Congenital microtia, Ear canal

## Abstract

**Objective:**

To investigate the role of MRI in the diagnosis and classification of fetal microtia.

**Methods:**

Ninety-five fetuses with suspected microtia based on ultrasound and MRI performed within 1 week were enrolled in this study. The diagnosis based on MRI was compared with postnatal diagnosis. Among the microtia cases suspected on the basis of MRI, mild and severe cases were further classified. In addition, external auditory canal (EAC) atresia was evaluated by MRI in 29 fetuses with a gestational age > 28 weeks, and the accuracy of MRI in the diagnosis and classification of microtia was determined.

**Results:**

Of 95 fetuses, 83 were considered to have microtia on the basis of MRI, 81 were confirmed to have microtia, and 14 were found to be normal according to postnatal diagnosis. Among 190 external ears in 95 fetuses, 40 ears were suspected to have mild microtia, and 52 ears were suspected to have severe microtia on the basis of MRI. According to the postnatal diagnosis, mild and severe microtia were confirmed in 43 and 49 ears, respectively. Among the 29 fetuses with a gestational age > 28 weeks, 23 ears were suspected to have EAC atresia according to MRI and 21 ears were ultimately confirmed to have EAC atresia. The accuracy of MRI in diagnosing microtia and EAC atresia was 93.68% and 93.10%, respectively.

**Conclusion:**

MRI shows good performance in diagnosing fetal microtia and has the potential to evaluate its severity on the basis of classification and EAC status.

**Clinical relevance statement:**

This study was aimed at investigating the role of MRI in the diagnosis and classification of fetal microtia. MRI shows good performance and can help evaluate microtia severity and EAC atresia, thus allowing for better clinical management.

**Key Points:**

*• MRI is a useful adjunct to prenatal ultrasound.*

*• MRI has a higher accuracy rate than ultrasound in diagnosing fetal microtia.*

*• The accurate classification of fetal microtia and the diagnosis of external auditory canal atresia through MRI may help guide clinical management.*

**Supplementary information:**

The online version contains supplementary material available at 10.1007/s00330-023-09816-5.

## Introduction

Fetal microtia is a common congenital anomaly of the external ear, with an incidence of 0.83–17.4 per 10,000 births worldwide [1, 2]. Microtia can also occur with genetic conditions affecting multiple body systems, such as Down, Turner, or trisomy 18 syndromes [1–5]. Therefore, early diagnosis of microtia may aid in the discovery of concurrent malformations [5, 6]. In addition, microtia in infants is always accompanied by conductive hearing loss on the affected side or both sides [3, 7, 8]. Hearing loss in only one ear can negatively affect future performance in school [9]. Therefore, early in utero diagnosis of fetal microtia is essential.

Microtia is graded according to Tsang’s 2016 classification [8] and is divided into subgroups according to severity (mild: grades I and II; severe: grades III and IV). Treatment for babies with microtia depends on the classification or severity of the condition. Currently, ultrasound (US) remains the first choice for prenatal diagnosis. However, US can be inconclusive in diagnosing microtia in fetuses with advanced gestational age (GA) or those in unfavorable positions, and in mothers with oligohydramnios, excessive obesity, or multiple gestations [6, 10, 11]. The detection rate of the fetal external ear by US has been reported to be 79.98%; however, this rate is only 50.73% after a GA of 36 weeks [6]. Furthermore, US can only partially evaluate the status of the external auditory canal (EAC). Nevertheless, EAC atresia is closely associated with hearing loss in children and is also an important indicator requiring evaluation before external ear reconstruction [12, 13]. For cases of severe microtia, particularly in patients with accompanying EAC atresia, surgical EAC repair is required. However, this procedure is relatively complex and challenging [7, 14]. Therefore, accurate preoperative assessment of microtia classification and determination of EAC status would enable better prenatal diagnosis and treatment.

Fetal MRI, has been used for the diagnosis of fetal abnormalities as a useful adjunct to US. By providing multi-directional and multi-parameter imaging, MRI can be used to assess ear size, morphology, and position, thereby revealing associated congenital malformations [15, 16]. Furthermore, MRI can delineate and measure EAC status, and can enable determination of microtia type. Moreira has reported that EAC can be identified as early as 26 weeks’ GA, and most cases (59%) can be identified after 29 weeks’ GA [17]. Notably, to our knowledge, no comprehensive studies have assessed the use of MRI for the diagnosis and classification of fetal microtia, and further evaluated EAC status.

In the present study, we aimed to investigate the potential role of MRI in the prenatal diagnosis and classification of fetal microtia. Our ultimate goal was to evaluate microtia defect severity and EAC status, to provide a reliable theoretical basis for better prenatal counseling and early management decision-making.

## Materials and methods

### Patients

The present study was approved by the Ethics Committee of Women’s Hospital, Zhejiang University School of Medicine (ethical approval no. IRB-20220075-R). Informed consent was provided by each participant included in the study.

From May 1, 2015, to May 31, 2021, a total of 104 fetal cases in which US indicated small or irregular external ear shape, in singleton pregnancies, were enrolled in our study. The US examinations were performed by two experienced ultrasound physicians (N.Y. and J.Q.) using Voluson 730D (GE) or Voluson E8 (GE) systems with a 3.5–6 MHz convex probe. No specific imaging requirements were provided, except that operators were asked to visualize the defects by using 3D surface rendering.

Of the 104 cases that underwent MRI examinations, six were excluded because of movement artifacts, and three were excluded because the external ears were covered by the uterine wall or placenta and could not be reconstructed by three-dimensional visualization, thus leaving 95 participants. All pregnant women successfully completed the MRI scanning within 1 week, and the average total scanning time was 27.19 ± 4.03 min. The average postprocessing time of three-dimensional volume rendering (3DVR) was 8 min (range, 6–12.5 min).

### MRI protocol

All scans were performed with a 1.5-T scanner (Signa HDxt; GE Healthcare) with an eight-channel cardiac coil. The pregnant women were placed in supine or lateral recumbent position while breathing smoothly. No sedation or contrast agents were administered.

First, a single-shot fast spin echo T2-weighted imaging (SSFSE-T2WI) sequence was used to image the fetal body. Subsequently, fast imaging with a steady-state acquisition (FIESTA) sequence was performed to scan the fetal head and ear in the axial, coronal, and sagittal planes. A 3D-FIESTA sequence with breath-hold was used to evaluate the fetal external ear and EAC. All data were then transferred to the imaging post-processing workstation (AW 4.6, GE) for 3DVR. The scanning parameters are shown in Table 1.

**Table 1:** MRI scanning parameters

MRI sequence	TR (ms)	TE (ms)	Bandwidth (Hz)	Thickness (mm)	Slice gap (mm)	FOV (mm^2^)	Flip angle
SSFSE-T2WI	2400	134	20.8	2.0	0	380 × 380	60°
FIESTA	3.6	1.7	80	2.0	0	380 × 380	55°
3D-FIESTA	3.5	1.5	41.7	2.0	—	400 × 400	60°

*Abbreviations*: *3D-FIESTA* three-dimensional fast imaging employing steady-state acquisition, *FIESTA* fast imaging employing steady-state acquisition, *FOV* field of view, *TE* echo time, *TR* repetition time

### MRI analysis

All MRI images, including 3DVR images based on the 3D-FIESTA sequence, were reviewed and evaluated by two experienced radiologists (X.Z. and K.L.). Discrepancies were resolved by consensus.

**Fig. 1:**
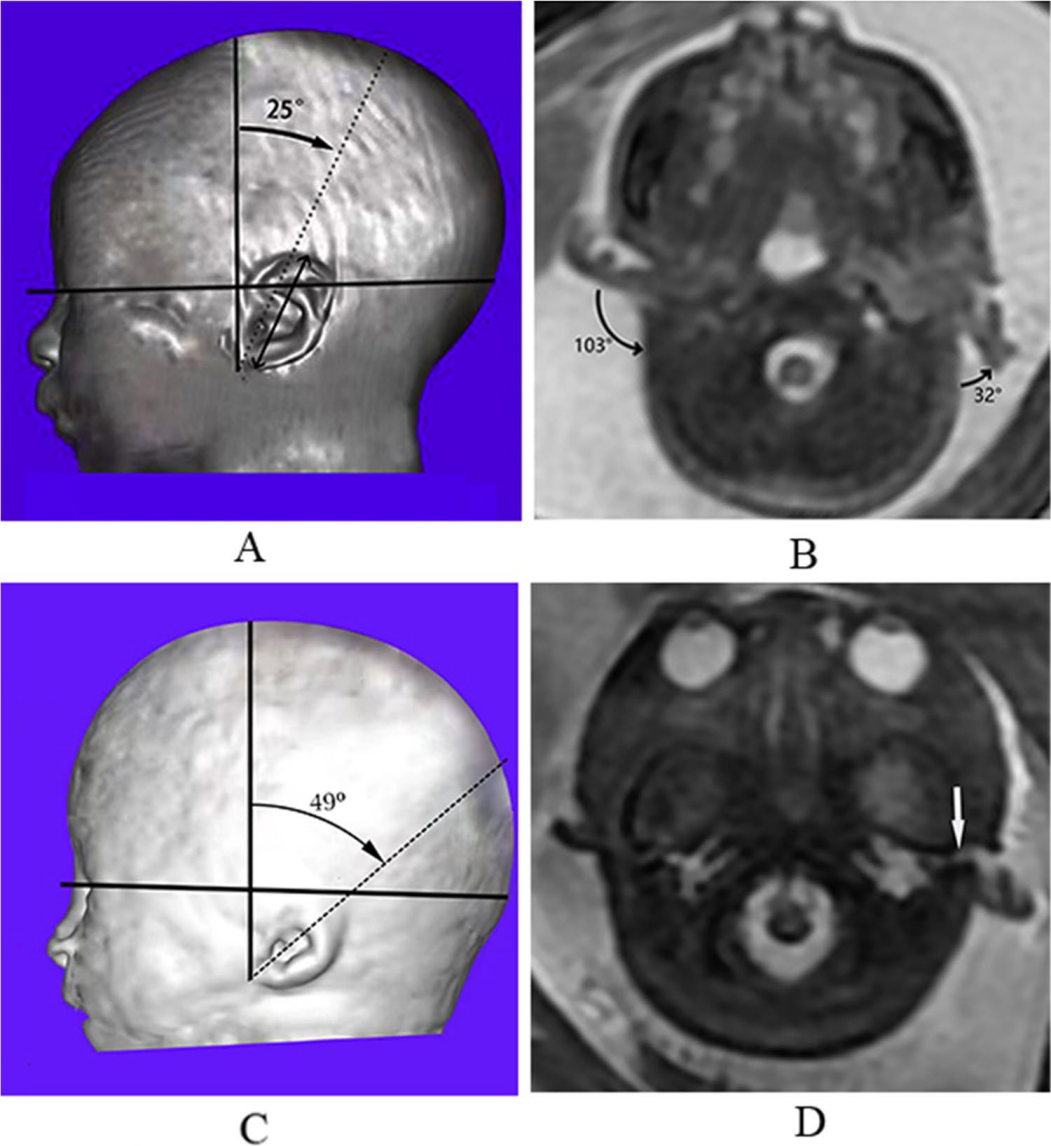
**A** The normal external ear positioned superior to the horizontal line in a 3DVR image of a fetal head; the horizontal line connects the outer canthus of the eye to the occipital protuberance. The ear rotation angle was determined by two lines, (i) the vertical line, which is perpendicular to the line connecting the outer canthus of the eye to the occipital protuberance, and (ii) the long axis of the external ear. A rotation angle < 30° is considered normal. **B** Auriculocephalic angle was defined as the angle subtended from the posterior aspect of the auricle to the mastoid plane of the skull. An angle exceeding 45° is considered abnormal. The left normal auriculocephalic angle was 32°, and the right abnormal auriculocephalic angle was 103°. **C** The external ear positioned below the horizontal line; the horizontal line connects the outer canthus of the eye to the occipital protuberance, i.e., a low-set ear. The ear rotation angle was measured to be 49°. **D** Axial T2WI showing hyperintensity of the left normal external auditory canal (EAC) on T2WI (white arrow), because the EAC was filled with amniotic fluid. The right ear presented microtia complicated with EAC atresia. EAC, external auditory canal; GA, gestational age; T2WI, T2 weighted imaging

The normally developed fetal ear is a fan-shaped, shell-like structure [11]. The long axis of the fetal ear is angled posteriorly upward (Fig. 1A). In single-shot fast spin-echo T2-weighted images (SSFSE-T2WI) and/or FIESTA sequences, normal external ears, as compared with the fetal musculature, appear as isointense structures with normal size, position, and angle; the EAC is seen as a small canal filled with hyperintense amniotic fluid connecting the fetal middle ear and the amniotic sac in SSFSE-T2WI and FIESTA sequences.

**Fig. 2:**
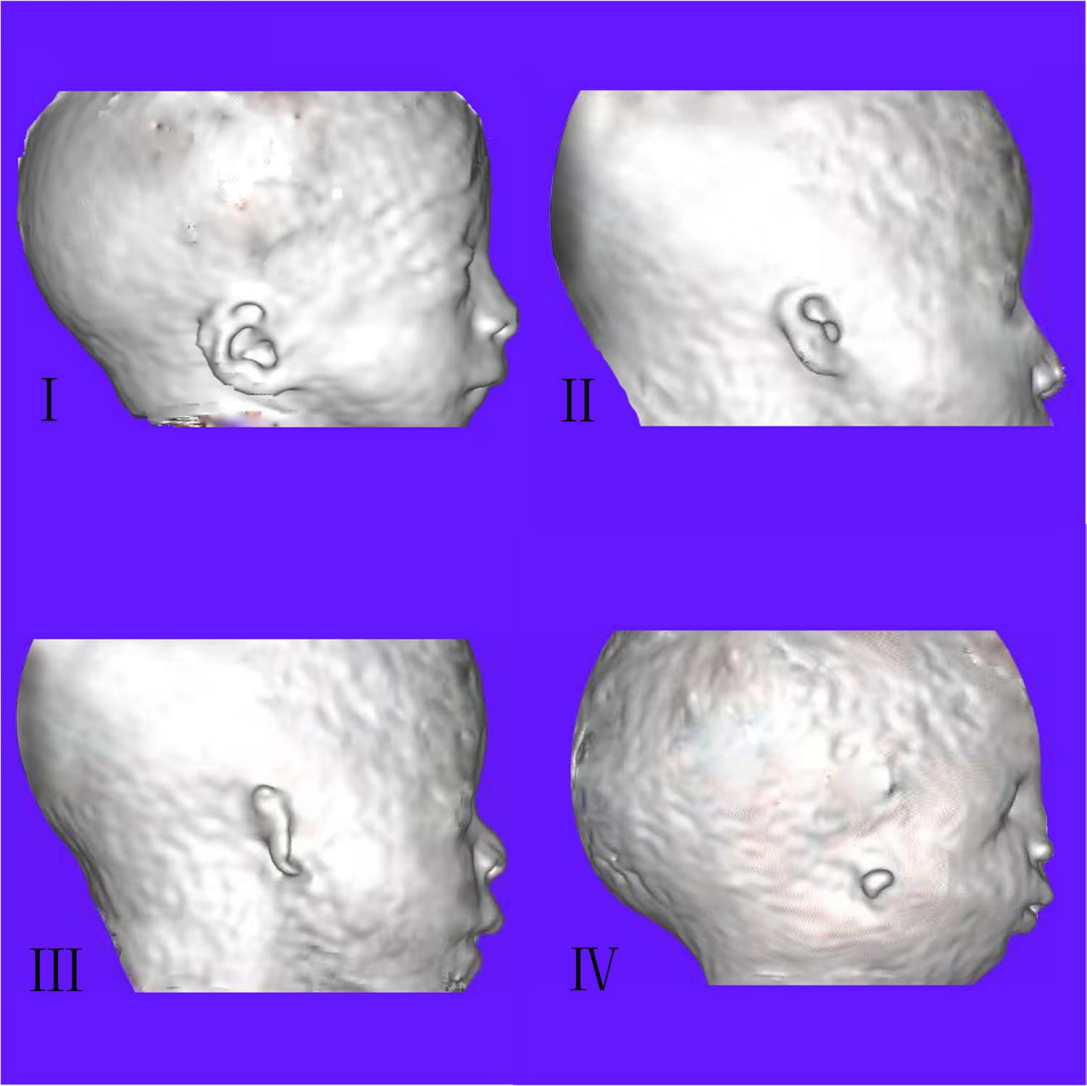
Fetuses with mild or severe microtia in 3DVR images. Mild microtia (grades I and II): In grade I, the ear is slightly smaller than normal, and the upper part is slightly abnormal in shape. In grade II, the ear is clearly smaller than normal, and the upper part anatomic subunits are deficient. Severe microtia (grades III and IV): In grade III, the ear presents as “peanut ear,” in which the upper half of the pinna is formed by disorganized cartilage, and the lower part has a malformed earlobe. In grade IV, the ear is almost invisible and forms a small “vegetation-like” structure. This fetus had micrognathia as a complication

The MRI features were as follows: (1) short ear length (EL); (2) low ear position; (3) orientation of the fetal external ear (auriculocephalic angle and ear rotation angle); (4) abnormal morphology; and (5) EAC atresia. Short EL was defined as two standard deviations below the mean value for GA [18, 19]; the measurement of EL is shown in Fig. 1 A. Low ear position was defined when the ears were positioned below the horizontal line from the outer canthus of the eye to the occipital protuberance [20] (Fig. 1A). For orientation of the fetal external ear, the auriculocephalic angle was defined as the angle subtended from the posterior aspect of the auricle to the mastoid plane of the skull [21], and an angle exceeding 45° was considered abnormal [22] (Fig. 1B). Ear rotation angle was determined by two lines, as illustrated by Fig. 1 C, and an angle exceeding 30° was considered abnormal [23]. Abnormal morphology included a sausage or peanut ear appearance or cupped shape ears [1, 2, 14, 19, 24]. According to the literature, EAC canalization is completed during the seventh month of fetal development [5]. Thus, EAC atresia in our study was defined by complete aplasia of the EAC with a bony atretic plate after a GA of 28 weeks [17]. EAC atresia was observed as an absence of hyperintense T2WI amniotic fluid in the EAC, which was replaced by soft or bony tissue and appeared as an isointense signal (Fig. 1D). The grading of microtia (grades I–IV) in our study was based on criteria by reported by Tsang in 2016 [8], wherein grades I and II are classified as a mild microtia subgroup, and grades III and IV are classified as a severe microtia subgroup (Fig.2). According to these standards, the average values of the two radiologists’ measurements were recorded as the final values of the EL, auriculocephalic angle, and ear rotation angle.

### Follow-up

Outcomes for all fetuses were followed up until birth or induced labor, through an interview at the clinic or by telephone. Postnatal diagnosis was determined through newborn physical examination or fetal autopsy, and the results were compared separately with the intrauterine imaging findings. All microtia types were recorded either as isolated findings or findings associated with other structural malformations.

### Statistics

Statistical analyses were performed in PASW statistics software (version 18.0; IBM Inc.). The intraclass correlation coefficient was used to assess interobserver agreements in the measurement of EL, auriculocephalic angle, and the rotation angle of microtia. Normally distributed continuous variables are presented as mean values ± SD, and categorical variables are presented as counts (percentage). With the postnatal diagnosis as the reference standard, the sensitivity, specificity, positive predictive value (PPV), negative predictive value (NPV), and accuracy of each index were calculated.

## Results

### Patient characteristics

A total of 95 cases were included in our study. The mean maternal age was 29.76 ± 4.76 years, and the maternal gravidity and parity were 1 (1.0–3.0) and 0 (1.0–2.0), respectively. The mean GA at US diagnosis was 25.97 ± 3.33 weeks, the mean GA at MRI diagnosis was 26.47 ± 3.41 weeks, and no statistical differences in GA were observed between US and MRI (*p* = 0.30), as shown in the Supplementary Table. Eventually, 55 of the pregnant women decided to continue the pregnancy, whereas 40 pregnant women chose to terminate the pregnancy.

### Diagnostic accuracy of microtia on MRI

The 95 microtia cases suspected on the basis of US comprised 88 cases of unilateral microtia and seven cases of bilateral microtia. However, in the same patients, MRI identified 83 cases, comprising 74 unilateral and nine bilateral cases; 12 fetuses diagnosed by US had normal MRI findings. Two of 88 fetuses were suspected to have unilateral microtia by US, and the contralateral ear was also suspected to be abnormal, according to MRI. On the basis of newborn physical examination or fetal autopsy, there were 72 confirmed cases of unilateral microtia, nine cases of bilateral microtia, and 14 fetuses with normal findings. A total of 79 cases were accurately diagnosed by MRI, including 70 with unilateral microtia and nine with bilateral microtia; four cases of unilateral microtia were misdiagnosed, and two cases were missed by MRI. The sensitivity, specificity, PPV, NPV, and accuracy of MRI in the diagnosis of fetal microtia were 97.53% (79/81), 71.43% (10/14), 95.18% (79/83), 83.33% (10/12), and 93.68% (89/95), respectively.

### Classification of fetal microtia on MRI

Of 190 external ears in 95 microtia cases suspected on the basis of US, prenatal MRI reaffirmed 92 microtia cases that were further graded. A total of 40 ears had mild microtia, and 52 ears had severe microtia, according to MRI. On the basis of newborn physical examination or fetal autopsy, 43 ears with mild microtia and 49 ears with severe microtia were finally confirmed. Of these, 81 ears were accurately diagnosed through MRI: 34 with mild microtia and 47 with severe microtia. On /MRI, five cases of mild microtia were misdiagnosed as severe microtia, two cases of severe microtia were misdiagnosed as mild microtia, four normal ears were misdiagnosed as having mild microtia, and four cases of mild microtia were missed (Table 2). As shown in Table 3, the sensitivity, specificity, PPV, NPV, and accuracy of MRI were 79.07% (34/43), 95.92% (141/147), 85.00% (34/40), 94.00% (141/150), and 92.11% (175/190), respectively, for mild microtia, and were 95.92% (47/49), 96.45% (136/141), 90.38% (47/52), 98.55% (136/138), and 96.32% (183/190), respectively, for severe microtia. The diagnostic sensitivity was higher for severe microtia than mild microtia (*p* = 0.01). Overall, MRI showed a high diagnostic accuracy for both mild and severe microtia; more importantly, it had better diagnostic sensitivity for fetal severe microtia.

**Table 2:** MRI diagnosis and postnatal results of 190 external ears with possible microtia

Prenatal MRI diagnosis	Postnatal diagnosis
Mild microtia (*n* = 40)	Mild microtia (*n* = 34) Severe microtia (*n* = 2) Normal (*n* = 4)
Mild microtia with EAC atresia* (*n* = 6)	Mild microtia with EAC atresia (*n* = 3) Severe microtia with EAC atresia (*n* = 2) Normal EAC (*n* = 1)
Severe microtia (*n* = 52)	Severe microtia (*n* = 47) Mild microtia (*n* = 5)
Severe microtia with EAC atresia* (*n* = 17)	Mild microtia with EAC atresia (*n* = 2) Severe microtia with EAC atresia (*n* = 13) Normal EAC (*n* = 2)
Normal ear (*n* = 98)	Normal ear (*n* = 94) Mild microtia (*n* = 4)
EAC atresia* (*n* = 0)	Mild microtia with EAC atresia (*n* = 1)^§^

^*^In fetuses with a gestational age > 28 weeks

^§^This case was missed by prenatal diagnosis

**Table 3:** Diagnostic performance measures of MRI in diagnosing microtia, its subtypes and external auditory canal atresia

Classification	SEN (95 CI, %)	SPE (95 CI, %)	PPV (95 CI, %)	NPV (95 CI, %)	ACC (95 CI, %)
Microtia^§^	97.53 (91.36–99.70)	71.43 (41.90–91.61)	95.18 (89.61–97.84)	83.33 (55.01–95.34)	93.68 (86.76–97.65)
Mild microtia	79.07 (63.96–89.96)	95.92 (91.33–98.49)	85.00 (71.83–92.64)	94.00 (89.75–96.56)	92.11 (87.31–95.51)
Severe microtia	95.92 (86.02–99.50)	96.45 (91.92–98.84)	90.38 (79.87–95.70)	98.55 (94.59–99.62)	96.32 (92.56–98.51)
EAC atresia*	95.24 (76.18–99.88)	91.89 (78.09–98.30)	86.96 (69.17–95.19)	97.14 (83.36–99.57)	93.10 (83.27–98.09)

*ACC* accuracy, *CI* confidence, *EAC* external auditory canal, *NPV* negative predictive value, *PPV* positive predictive value, *SEN* sensitivity, *SPE* specificity

^§^Diagnostic performance measures were evaluated in 95 fetuses; other indices were evaluated in 190 ears

^*^In fetuses with gestational age > 28 weeks

### Diagnostic accuracy of EAC atresia on MRI

Because the EAC is well-developed during the seventh month of gestation [5], 29 fetuses with GA greater than 28 weeks were included in the evaluation of EAC. A total of 23 ears were suspected to have EAC atresia according to MRI: 6 with mild microtia and 17 with severe microtia. On the basis of newborn physical examination or fetal autopsy, 21 ears were confirmed to have EAC atresia: six with mild microtia and 15 with severe microtia. One case of mild microtia was misdiagnosed, and one case was missed. Two cases of severe microtia were misdiagnosed, and no cases were missed by MRI. The diagnostic sensitivity, specificity, PPV, NPV, and accuracy of MRI in diagnosing EAC atresia were 95.24% (20/21), 91.89% (34/37), 86.96% (20/23), 97.14% (34/35), and 93.10% (54/58), respectively. Specifically for mild microtia, the diagnostic sensitivity and accuracy of MRI in diagnosing EAC atresia were 83.33% (5/6) and 71.43% (5/7); for severe microtia, these values were 100.00% (15/15) and 88.24% (15/17), respectively. No differences in the diagnostic accuracy of EAC atresia were observed between mild and severe microtia cases (*p* = 0.33).

### Other associated structural malformations

A total of 19 cases had complications of other malformations. The rates of microtia complication involving associated malformations in fetuses with unilateral microtia and bilateral microtia were 18.06% (13/72) and 66.67% (6/9), respectively, and a clear statistical difference was observed between groups (*p* = 0.001; specific information summarized in Table 4). These data suggest that if bilateral external ears anomalies are suspected, a complete fetal MRI examination should be performed to support further clinical management.

**Table 4:** Associated structural malformations of unilateral and bilateral microtia

	Cases	Associated structural malformations
Unilateral microtia (*n* = 13)	Case 1	Orofacial cleft, cleft palate, micrognathia
	Case 2	Orofacial cleft, cleft palate, micrognathia
	Case 3	Horseshoe kidney, dysplasia of right kidney, disorder of cervical vertebra alignment
	Case 4	Orofacial cleft, micrognathia
	Case 5	Cleft palate, mandibular dysplasia
	Case 6	Disorder of cervical vertebra alignment, varus foot
	Case 7	Cleft lip
	Case 8	Isolate cleft palate
	Case 9	Polydactyly
	Case 10	Ventricular septal defect
	Case 11	Small stomach
	Case 12	Hydronephrosis
	Case 13	Hypoplasia right foot
Bilateral microtia (*n* = 6)	Case 14	Pierre Robin syndromes
	Case 15	Pierre Robin syndromes
	Case 16	Orofacial cleft, cleft palate, micrognathia
	Case 17	Holoprosencephaly, nasal malformation, mandibular dysplasia, cleft palate
	Case 18	Dandy-Walker malformation, tetralogy of Fallot, syndactyly
	Case 19	Complete transposition of the great arteries, ventricular septal defect, hydronephrosis

## Discussion

In this study, we demonstrated that prenatal MRI can be used for the diagnosis of fetal microtia and classification of microtia with satisfactory accuracy. More importantly, MRI showed better diagnostic ability than US in the evaluation of EAC atresia, to guide patient counseling regarding postnatal management.

Currently, US is extensively used for prenatal diagnosis. However, US results can be inconclusive in certain cases, such as those with advanced GA, oligohydramnios, multiple gestations, or acoustic shadows from surrounding bony structures [10, 11, 25]. One study has demonstrated that the xMatrix probe provides more detailed 3D image resolution of the fetal ear than a conventional mechanical probe. However, the highest percentage of ear visualization was 82.5%, and the study examined only normal fetal ears [25]. To date, no comprehensive studies have examined the use of US for diagnosing fetal microtia. When US efficiency is limited, MRI can serve as an effective supplemental method for the measurement and evaluation of fetal external ears with superior soft-tissue resolution and contrast [15]. Milic et al [16] have reported a case of trisomy 22 with multiple prenatal malformations; however, the diagnosis of bilateral microtia was missed in the US of this fetus, which was supplemented through MRI. Three-DVR images of MRI provided a detailed visualization of the fetal ear, including the helix, antihelix, tragus, antitragus, concha, and lobule. In the present study, two cases of US indicated suspicion of unilateral microtia, whereas MRI indicated a mild malformation in the other ear, which was ultimately confirmed by postnatal diagnosis. Hence, our data suggest that MRI renders fetal microtia with better definition, with more satisfactory diagnostic accuracy than US.

To date, no studies have focused on the classification of fetal microtia by using US or MRI. Managing microtia cases is challenging [8, 26], particularly because the clinical symptoms and treatment of microtia in postnatal infants vary according to the classification and severity. Accurate evaluation of microtia classification before surgery is essential for treatment success [8, 14, 24]. We observed that fetal microtia exhibited the following features on MRI, which enabled classification with high accuracy: (i) short EL, (ii) low position, (iii) protruded ear, (iv) posteriorly rotated ear, (v) abnormal morphology, and (vi) EAC atresia. Classic microtia cases have obvious small volume and morphological abnormalities, which are fairly easy to diagnosis. However, when the morphological changes in mild microtia or even diminished ear length are not immediately apparent, the diagnosis must be assisted by the assessment of other features of microtia. In our study, MRI had a relatively high accuracy in diagnosing mild and severe fetal microtia, and a high sensitivity in diagnosing severe microtia, particularly with 3DVR imaging, thus indicating that the classification of fetal microtia on MRI is both accurate and valuable in predicting prognosis and comparing treatment option [5].

Here, we provided the first evidence that MRI not only can distinguish mild from severe microtia, but can also be used to evaluate EAC atresia, thus aiding in early management decisions before delivery. One study has found that better developed EAC is correlated with better hearing levels [13]. Even mild microtia associated with EAC atresia can cause hearing loss; consequently, prenatal diagnosis of EAC atresia is necessary and important. The ability of US to exclude temporal bone pathology in utero is limited [17]. To date, only one study, by Wei and colleagues [6], has reported the case of one fetus with unclear EAC screened by US. Because soft tissue has good resolution on MRI, and the normal EAC is filled with amniotic fluid that is hyperintense on SSFSE-T2WI or FIESTA sequences, EAC can be clearly observed against the isointense adjacent soft tissue. Several detailed MRI studies have been published regarding the development of EAC during gestation; however, the diagnostic performance of MRI for EAC atresia has not been reported [10, 16, 17]. A study by Moreira has indicated that EAC can be identified as early as 26 weeks’ GA [17]. In contrast, two cases of EACs were identified as early as 23 weeks’ GA in our study and were later confirmed by postnatal diagnosis. Our earlier detection might have been because we used 2 mm-thickness scanning in transverse and coronal planes, whereas the prior study used a scanning thickness of 3–4 mm and consequently might have missed the thin, narrow EAC. In the present study, MRI had high accuracy in diagnosing mild or severe microtia with EAC atresia in fetuses at > 28 weeks’ GA. Moreover, in severe microtia cases, the sensitivity and NPV of EAC atresia were both 100%. This higher diagnostic accuracy has the potential to facilitate subsequent clinical consultations and treatments for fetal microtia.

Microtia is frequently accompanied by other structural malformations. Previous studies have reported that microtia may be isolated or associated with other deformities, chromosomal abnormalities, or various syndromes [27–29]. Harris [28] has suggested that facial clefts and cardiac defects are the most common associated malformations (30% each), followed by anophthalmia or microphthalmia. Reiko [29] has suggested that 32.7% have an accompanying malformation, whereas 13.3% have associated syndromes (e.g., craniofacial microsomia or Treacher Collins syndrome). Hartzell [30] has reported that, although the proportion of fetuses with bilateral microtia is only 10%, the likelihood of combined malformation is significantly higher than that in fetuses with unilateral microtia. Our findings are consistent with those of these prior studies, thus confirming that fetuses with bilateral microtia are more likely to have other structural malformations than fetuses with unilateral microtia (66.67% vs. 18.06%, *p* = 0.001).

## Limitations

This study has several limitations. First, it was a retrospective study in only a single hospital center; therefore, bias was inevitable. Second, owing to the influence of magnetic resonance technology, we did not evaluate the middle and inner ear in fetuses in utero. Third, EAC malformation can also include EAC stenosis, but because no standard exists for stenosis in the fetal period, this aspect was not evaluated in our study. Finally, regional and race differences were not considered in our study.

## Conclusion

MRI is a useful tool for fetal microtia assessment. More importantly, MRI can help assess fetal microtia severity and EAC atresia, thus allowing for better prenatal counseling and early management decisions.

### Supplementary Information

Below is the link to the electronic supplementary material.

Supplementary file1 Supplementary table 1 Note: Normal-distributed variables were presented as mean values ± SD, skewed-distributed variables were presented as median (min-max). (PDF 97 KB)

## Abbreviations

3DVR: Three-dimensional volume rendering
EAC: External auditory canal
EL: Ear length
GA: Gestational age
MRI: Magnetic resonance imaging
NPV: Negative predictive value
PPV: Positive predictive value

## Declarations

### Guarantor

The scientific guarantor of this publication is Baohua Li.

### Statistics and biometry

No complex statistical methods were necessary for this paper.

### Methodology

- Retrospective
- Diagnostic or prognostic study
- Performed at one institution
