# Supplementary material for: The role of MRI in the prenatal diagnosis and classification of fetal microtia
Source: Eur Radiol. 2023 Jun 14;33(11):7707–15. doi: 10.1007/s00330-023-09816-5 (PMC10598147; doi:10.1007/s00330-023-09816-5)
Supplement: Supplementary file 1 — Supplementary file1 Supplementary table 1 Note: Normal-distributed variables were presented as mean values ± SD, skewed-distributed variables were presented as median (min-max). (PDF 97 KB) [file 330_2023_9816_MOESM1_ESM.pdf]

Supplementary table 1: Baseline characteristics of mothers at the time of MRI or ultrasound examination

Characteristics	Value
**Number of participants**	95
**Conception method**	
Natural conception	86
IVF	9
**Maternal age** (years)	29.76 ± 4.76 (20 – 44)
**Gravidity**	1 (1.0 – 3.0)
**Parity**	0 (0 – 2.0)
**GA at US diagnosis** (weeks)	25.97 ± 3.33 (18 – 39)
**GA at MRI diagnosis**	26.47 ± 3.41 (19 – 39)
**Delivery method**	
Vaginal delivery	39
Cesarean delivery	16
Induced labor	40
